# 565 Impact of Municipal Ordinances on Firework-Related Injuries

**DOI:** 10.1093/jbcr/irac012.193

**Published:** 2022-03-23

**Authors:** Austin T Mefford, Robert P Duggan, Katie Kirk, Alen Palackic, Ludwik K Branski

**Affiliations:** University of Texas Medical Branch, Galveston, Texas; University of Texas Medical Branch, Galveston, Texas; University of Texas Medical Branch, Galveston, Texas; University of Texas Medical Branch, Galveston, Texas; University of Texas Medical Branch, Galveston, Texas

## Abstract

**Introduction:**

There is significant heterogeneity in firework-related legislation across the country, with some states outright banning sales, possession, and use. Others restrict the dates and types of fireworks that can be purchased. Municipalities often adopt firework ordinances that apply within their city limits, pushing the sale and use into unincorporated areas or permitting easy access to illegal fireworks for transport into city limits. We seek to understand how effective firework ordinances are at preventing firework injuries. We hypothesize lax municipal ordinances will have a limited effect on firework-related injuries.

**Methods:**

Two time periods where the commercial sale of fireworks is legal were identified, and we reviewed patients presenting during those windows for the acute management of firework-related burns. This corresponds to June 24 – July 11 and December 20 – January 8. Patient demographics and burn outcomes were collected. Socioeconomic status was determined using a standardized scale (0-100) that incorporates Census data, with higher scores reflecting more disadvantaged communities. Legality was determined by a review of municipal ordinances and patient residence.

**Results:**

Thirty-five patients were identified between December 2016 and January 2021. More injuries occurred around July 4^th^ compared to New Year’s (54% vs. 46%). The cohort was predominantly men (77%) with an average age of 29 years. Patients most commonly came from suburban areas (34.3%), compared to rural (25.7%), urban (20%), or small towns (17.1%). Alcohol use at the time of injury was reported in 14% of cases. Explosive fireworks (e.g., mortar shells) (63%) were more common than sparklers. Hands were the most frequently injured area (83%), but no amputations, traumatic or surgical, occurred. Eye injuries occurred in 17.1% of patients, but no long-term damage to vision was sustained. Eight patients (23.9%) required inpatient management, and six (17.1%) required operative management. Split thickness skin grafting and local tissue rearrangement were the extent of the operations performed. One patient required neurosurgical intervention after a mortar shell detonated adjacent to their cranium, representing the lone fatality in the cohort. There was no significant difference in the number of legal (18) and illegal (17) firework injuries. Socioeconomic status was not different between legal and illicit groups (46 vs. 54, p = 0.101).

**Conclusions:**

A substantial number of patients sustained firework-related injuries in municipalities where fireworks are banned, giving the impression that current ordinances are ineffective. Public health officials and legislatures may consider the more widespread implementation of regulations or increased penalties to combat firework-related injuries.

